# Unraveling EFL Teacher Motivation for Pursuing a Master of Education Degree in the Chinese Context

**DOI:** 10.3390/bs15040473

**Published:** 2025-04-06

**Authors:** Lixiang Gao, Honggang Liu, Zizheng Shen

**Affiliations:** 1 School of Foreign Languages, Fuyang Normal University, Fuyang 236037, China; gorlix123@fynu.edu.cn; 2 School of Foreign Languages, Soochow University, Suzhou 215008, China; zzshen123@stu.suda.edu.cn

**Keywords:** motivation for pursuing Ed.M., motivational factors, Chinese EFL teachers

## Abstract

In recent years, the topic of language teacher motivation has garnered significant attention within the realm of language teacher psychology. Researchers have delved into various aspects, including teachers’ commitments to the teaching career, teachers’ teaching motivation, and teachers’ professional development motivation. Nevertheless, the motivation of English as a Foreign Language (EFL) teachers to engage in ongoing in-service learning, particularly the pursuit of a Master of Education (Ed.M.) degree, has received comparatively less scrutiny. To bridge this gap, the present study adopted Boshier’s Education Participation Scale (EPS) and Liu’s seven-dimensional motivation framework to explore the motivation of 529 Chinese EFL teachers in their quest for an Ed.M. degree. Utilizing Exploratory Structural Equation Modeling (ESEM), the analysis revealed seven types of key motivation: cognitive interest, social responsibility, academic information acquisition, academic achievement acquisition, school context, rival demand, and significant others. An examination of differences in EFL teacher motivation in terms of gender and school type showed that male teachers perceived significantly higher levels of cognitive interest and rival demand than female teachers did. And, teachers in regular schools reported significantly higher levels of significant others than those in key schools. We propose some future directions for EFL teacher motivation research.

## 1. Introduction

Teachers play a pivotal role in language education, and their relentless pursuit of professional growth is paramount to fostering excellence in language instruction. This pursuit necessitates a myriad of opportunities, including, but not exclusive to, being visiting scholars (Liu et al., 2020), participating in academic conferences (Borg, 2015), and embarking on master’s degree programs (Greene et al., 2020). The intricate motivation that guides teachers’ decisions through these avenues has garnered significant attention in recent scholarly studies (e.g., Fujita-Starck, 1996; Boeren et al., 2012; Gorozidis & Papaioannou, 2014; Appova & Arbaugh, 2018). Among these paths, pursuing an Ed.M. degree stands is a potent means. Because it not only can promote EFL teachers’ professional development but also helps them enhance their academic qualifications. As a result, many teachers choose this path. However, the reasons behind their decision vary, and research on this topic, especially within China’s unique educational system, remains limited.

China’s EFL context presents a critical yet underexplored research frontier. As the country with the largest EFL learner population, academic qualifications play a central role in career advancement, influencing promotions, salary increases, and institutional recognition. However, most research focuses on factors driving the pursuit of doctoral degrees (Tarvid, 2014; Fung et al., 2017). This leaves a gap in understanding how systemic and cultural factors, such as qualification-driven policies and Confucian values of lifelong learning, shape teachers’ motivations for pursuing an Ed.M. degree. Given the program’s potential to improve teaching quality on a large scale, this gap is especially pressing.

Furthermore, the interplay between internal and external motivations remains opaque. Some teachers pursue an Ed.M. for career mobility, while others seek personal fulfillment through enhanced pedagogical creativity or a stronger professional identity. Understanding this dynamic is essential for designing targeted incentives. For example, flexible study policies may support internally motivated teachers, while promotion-based frameworks could appeal to those driven by external rewards. Failure to address these nuances risks perpetuating a one-size-fits-all approach to professional development, potentially exacerbating burn-out in China’s high-pressure, exam-oriented EFL environment.

Therefore, it is imperative to delve into and validate the intricate motivation and inner workings of EFL teachers’ decisions to pursue an Ed.M. degree in China. This inquiry is not just theoretical; it has practical implications for policymakers allocating resources, institutions refining teacher support, and educators making informed career decisions. By exploring how cultural, systemic, and personal factors interact, this research can inform policies that integrate Ed.M. credentials into national accreditation standards, strengthen university–school partnerships, and create blended learning models. These findings may also contribute to academic discussions on teacher motivation, highlighting how credentialism and collectivist values shape professional development across different contexts.

To address these issues, this study examined the motivations of 529 in-service primary and middle school EFL teachers in China. These teachers pursued an Ed.M. degree part-time while working full-time. This research aims to provide new insights into teacher motivation, contributing to a deeper understanding of this critical topic.

## 2. Boshier’s Framework of Adult Education Participation Motivation

It is generally believed that the study on adult education participation motivation began with Houle (1961), who researched 22 continuing education participants in Chicago. Through these interviews, he identified three categories of motivation: goal orientation, activity orientation, and learning orientation. Goal orientation refers to adults seeing education as a way to achieve specific goals; activity orientation refers to adults finding things unrelated to learning content and activities in the learning environment; and learning orientation refers to adults learning purely for the sake of knowledge.

Based on Houle’s (1961) findings, Sheffield (1964) conducted a study among 453 adult education participants using a 58-item questionnaire to investigate their motivation to participate in education. The results revealed five dimensions of their motivation: learning orientation, desire–activity orientation, personal goal orientation, societal goal orientation, and need–activity orientation.

To test Houle’s (1961) typology of motivational orientation, Boshier (1971) designed an EPS containing 48 items and examined it among 233 adult education participants. Through factor analysis, four types of motivation factors were extracted, namely, other-directed professional advancement, educational preparedness, self-centredness versus altruism, and social contact. Boshier (1977) conducted a detailed analysis of the EPS and identified five key factors: escape/stimulation, professional advancement, social welfare, external expectations, and cognitive interest. This expanded the original four-dimensional EPS model (Boshier, 1971) into a five-dimensional one. By inviting EPS users to contribute their data, Boshier and Collins (1983) conducted a secondary analysis. Finally, the study extracted six factors: social contact, social stimulation (escape/stimulation factor), professional advancement, community service (social welfare), external expectations, and cognitive interest. Up to this point, the EPS evolved into a 40-item, six-dimensional framework (see Table 1), which remains widely recognized and frequently used by researchers. Since then, Boshier has continued to refine the EPS, expanding its application across different cultural and educational contexts (Boshier, 1991; Boshier et al., 2006; Boshier & Huang, 2010). His ongoing work has further solidified the EPS as a key tool in studying adult education participation motivation.

## 3. Teacher Motivation for Professional Development

Teacher motivation is a classical topic in teacher education research (Liu, 2020), one type of which is to explore why teachers pursue their professional development in different ways. Teachers’ professional development is defined as utilizing formal and informal learning opportunities (Wu & Liu, 2024) to deepen and extend their professional competence, such as knowledge, beliefs, motivation, and self-regulatory (Richter et al., 2011). Generally, formal learning opportunities (i.e., being visiting scholars and attending graduate courses) are preferred among teachers due to the structured learning environments with a specified curriculum. Teachers’ motivation for engaging in various developmental models reflects their orientations toward in-service professional education (Liu et al., 2020). Researchers in the field of teacher education in different contexts have made great efforts to explore teacher motivation for in-service professional development.

### 3.1. Teacher Motivation for Being Visiting Scholars

Being visiting scholars or attending a visiting scholar program means teachers can participate in short-term academic projects at home or overseas universities without undertaking teaching tasks. Some researchers explored the reasons why teachers chose to be visiting scholars. Liu et al. (2020) administered a questionnaire among 169 teachers to investigate their motivation for being visiting scholars in the Chinese context. Through an exploratory factor analysis, six motivational factors were identified: internal needs, stress relief, academic positioning, academic contact, academic symbolism, and policy support. Zhao and Liu (2022) explored 314 Chinese visiting scholars’ transformative learning experiences and identified three main themes. They were the cognitive, affective, and behavioral domains. The cognitive domain included teachers’ changes in perspective and understanding, such as their previously held or established assumptions. The affective domain encompassed teachers’ changes in motivational and emotional aspects, such as more enthusiasm for research and more confidence academically. The behavioral domain included teachers’ changes in research and teaching or professional practice, such as innovative educational research methods and research orientation. It can be seen that teachers choose to be visiting scholars for different reasons, and they can also benefit much from visits in different aspects. No matter what your motivation is, being a visiting scholar is one of the effective means of seeking professional development.

### 3.2. Teacher Motivation for Pursuing a Master’s Degree

The research on teachers pursuing master’s degrees has embraced more prevalence due to its well-recognized property. For example, Huang and Xu (2005) took 737 Chinese primary and secondary school teachers who were studying for a master’s degree in education as research subjects and used a questionnaire to explore their motivation for studying. The results found that their learning motivation could be divided into four types: career planning (reflecting on the occupation and work they are engaged in and considering changing careers), professional advancement (aiming to enrich their knowledge and improve their professional ability to better perform their work), external influence (being influenced by friends, colleagues, and other people around them or encouraged by the unit), and rival demand (to be in more competitive in promotion). Y. Li and Shao (2016) investigated 248 Chinese in-service teachers’ motivation for the pursuit of an Ed.M. degree. The results showed there were two types of motivation, which were internal and external motivation. The former included the need for self-growth and career re-planning, and the latter included future rival demand and external factor influence.

Greene et al. (2020) used a mixed-methods approach to investigate the motivational factors influencing 31 American teachers of various ages in their pursuit of a Master of Arts in Education (MAED). The study found that the primary motivation for teachers to pursue a master’s degree was extrinsic, including factors like the program’s duration, the desire for more professional opportunities, and attending an accredited university. In contrast, intrinsic motivations, such as a commitment to lifelong learning and loyalty to the university, were not the main drivers behind teachers’ decision to pursue the degree.

The aforementioned research provides a robust literature foundation and empirical research support in exploring the motivation of teachers engaging in professional development. Although these studies identified motivational factors for professional development and uncovered the intricate interplay of these factors, several limitations and gaps remain. Firstly, most of them failed to utilize a theoretical framework like Boshier’s EPS as a guide to portray the whole picture of in-service teachers’ motivation. By ignoring such a framework, these studies missed the opportunity to systematically examine how diverse factors interact and influence teachers’ decisions to participate in professional development activities. Secondly, the methodologies employed in these studies tend to be quantitative in nature, relying heavily on surveys and questionnaires. While these methods provide valuable data on a large scale, they may not capture the nuance and depth of teachers’ motivations, which are often complex and multifaceted. Qualitative approaches could offer richer insights into the personal and contextual factors that influence teachers’ decisions to engage in professional development. Thirdly, there is a lack of research on in-service EFL teachers’ motivational factors in China. Considering their heavy workload and trivial family affairs, factors that drive them to pursue Ed.M. are well worth exploring. Therefore, this study employed Boshier’s (1991) EPS and Liu’s (2015) seven-dimensional motivation framework to investigate primary and middle school EFL teacher motivation for pursuing an Ed.M. degree.

### 3.3. Theoretical Framework

Upon meticulously examining the aforementioned research, it can be concluded that delving into the motivation behind in-service teachers’ pursuit of an Ed.M. degree is a multidimensional exploration that promises to illuminate not just the individual hearts and minds of teachers but also the broader landscape of education itself, fostering a culture where lifelong learning is celebrated and nurtured. Therefore, this study employs Boshier’s (1991) EPS and Liu’s (2015) seven-dimensional motivation framework to investigate primary and middle school EFL teacher motivation for pursuing an Ed.M. degree. The framework proposes seven types of in-service teacher motivation:

1. Academic information acquisition: It means teachers’ relentless pursuit of cutting-edge academic insights, teaching resources, and expert mentorship. English teachers often face the hurdle of inadequate access to the academic information necessary to undertake research projects, let alone specialized guidance. Enrolling in a prestigious university presents a golden opportunity for them to immerse themselves in these invaluable resources, fostering a fertile ground for intellectual growth and research endeavors.
2. Rival demand: It alludes to teachers’ drive for professional advancement, promotion, social recognition, and the allure of honor. The path to title promotion for teachers is fraught with challenges, where the competition is intense and unforgiving. Without a distinct edge, teachers may risk falling behind in the race. Pursuing an Ed.M. degree serves as a strategic move, empowering teachers to enhance their skills, knowledge, and ultimately, their standing within the profession, enabling them to stay ahead of the curve.
3. Academic achievement acquisition: It signifies teachers’ aspiration to publish scholarly articles, secure research grants, or lay the groundwork for doctoral studies through master’s education. For primary and middle school EFL teachers, navigating the landscape of publishing and project funding can be daunting. However, these accomplishments are paramount for career advancement. Seeking an Ed.M. degree offers a viable solution, providing the necessary tools and support to overcome these obstacles and propel their academic careers forward.
4. Social responsibility: It underscores the noble impulse of teachers to disseminate the knowledge gained during their postgraduate studies among their peers, fostering collective improvement and enhancing the overall educational landscape. Beyond personal growth, teachers are driven by a sense of duty to contribute to the development of their colleagues, schools, and society at large. By doing so, they strive to realize their societal value and make a meaningful impact.
5. School context: It refers to teachers being attracted by the prestigious reputation and unique strengths of the university that they choose to attend. Teachers are often drawn to esteemed institutions, captivated by their renowned academic programs and prestigious status. Attending such universities fills them with a sense of pride and accomplishment, which they cherish deeply and proudly share with others when discussing their postgraduate experiences. This not only enhances their personal satisfaction but also adds luster to their professional profiles.
6. Significant others: It refers to the profound impact that classmates, colleagues, friends, and family members can have on teachers, serving as a catalyst for their decision to embark on an Ed.M. degree journey. As the saying goes, “No man is an island” and EFL teachers, too, are interconnected with the world around them. Their interactions with these significant figures inevitably lead to the exchange of ideas, encouragement, and inspiration. Positive reinforcement and experiences from these individuals can powerfully motivate teachers to pursue further education, ultimately choosing the path of an Ed.M. degree to enrich their professional lives.
7. Cognitive interest: It highlights teachers’ innate drive to refine their teaching prowess, research capabilities, and reflective practices through postgraduate studies. In an era of relentless progress, educators often find themselves navigating uncharted waters, challenged by rapid technological advancements like AI. To remain competitive, relevant, and effective in their roles, many teachers recognize the need for continuous learning and self-improvement. Consequently, they turn to Ed.M. programs to stay ahead of the curve, enhancing their skills and competencies to better equip themselves for the demands of the modern educational landscape.

Drawing upon this foundation, the present study mainly addresses three research questions:

1. What motivational factors influenced EFL teachers to pursue an Ed. M. degree?
2. What are the global and dimensional levels of EFL teacher motivation for pursuing an Ed.M. degree?
3. Are there any differences in EFL teacher motivation for pursuing an Ed.M. degree in terms of gender and school type?

## 4. Methodology

### 4.1. Research Participants

Convenience sampling was utilized to recruit teachers who are currently enrolled in or have completed their Ed.M. program. We selected three provinces from different regions of mainland China, which collectively account for approximately half of the country’s population. To ensure the relevance of our data, we established specific sampling criteria for participant selection, guided by the recommendations of Hair et al. (2019) and Rose et al. (2019). These criteria included (1) teachers who are currently pursuing or have completed their Ed.M. program; (2) foreign language teachers; and (3) teachers from primary and secondary schools. Our sample was considered reasonably representative of the national population of primary and secondary school teachers, as it encompasses a diverse range of teachers across gender, school types, and school locations (see Table 2).

A total of 529 primary and middle school English teachers from different parts of China participated in this research, among whom there were 61 male teachers (11.5%) and 468 female teachers (88.5%). The number of teachers in key schools is 342 (64.7%) and 187 (35.3%) in regular schools (see more in Table 2). There are 333 urban teachers (62.9%), 178 town teachers (33.6%), and 18 rural teachers (3.4%). In addition, four of them were invited to join the interview part to triangulate quantitative data.

### 4.2. Research Instruments

English Teachers’ Motivation for Pursuing an Ed.M. Degree Scale was utilized as the instrument in this study, which was revised from Boshier’s (1991) EPS and Liu’s (2015) Survey of Foreign Language Teachers’ Motivation for Pursuing an Ed.M. Degree. The 24-item questionnaire employed a six-point Likert scale ranging from 1 (completely does not fit my situation) to 6 (completely fits my situation). The qualitative data were collected using interviews. The main open-ended questions included the following: What were the reasons for you pursuing an Ed.M. degree? Describe your experience and the ways in which this experience impacted and changed your thinking.

### 4.3. Data Collection and Analysis

We employed R (version 4.3.3), Mplus (version 8.3), and SPSS (version 26.0) for quantitative data analysis. Quantitative data collection lasted nearly one month from April 2023 to May 2023. During this process, questionnaires were circulated through an online questionnaire system Wenjuanxing to teachers working at primary and middle schools in China. In total, 572 questionnaires were collected. After screening them based on answer time and distribution, 529 questionnaires were valid, with an effective rate of 92.48%.

After that, univariate and multivariate normal distribution tests were conducted on the data. The results show that the kurtosis and skewness values for each item are within the range of ±8 and ±3, respectively (see Table 3), indicating that each item in the scale exhibits univariate normal distribution (Kline, 2023). However, the test for multivariate normal distribution shows that the *p*-values for both multivariate kurtosis and skewness are less than 0.05, indicating that the data does not exhibit multivariate normal distribution (Mardia, 1974).

Then, ESEM was conducted to further validate Boshier’s (1991) and Liu’s (2015) motivation frameworks. Considering the confirmatory nature of this study and the non-normality of the data, we used the WLSMV estimation and target rotation. The WLSMV estimator is robust, as it does not assume normality in variables, making it the optimal choice for modeling categorical or ordinal data (Brown, 2006).

In addition, to verify and supplement the quantitative findings, qualitative interview data were collected in June 2023 and transcribed in textual form utilizing voice software (iFlyREC, version 2.0.1531), and then they were checked by the researchers and the interviewees. Some wrongly expressed sentences were appropriately modified, and relevant segments related to this study were extracted to supplement the quantitative data. This study conducted a simple coding of the basic information of the teachers who were interviewed, such as C-I-20230610, where C represents Teacher C, I represents the interview, and 20230610 represents the interview date of 10 June 2023.

## 5. Results and Discussion

### 5.1. Results of ESEM of EFL Teacher Motivation for Pursuing Ed.M.

The results of ESEM show that the values of CFI and TLI are both above 0.95 (CFI = 0.980, TLI = 0.956), the RMSEA is less than 0.7 (RMSEA = 0.062), and the SRMR is less than 0.05 (SRMR = 0.019), indicating that the model is acceptable (Alamer & Marsh, 2022). In addition, according to the criteria for evaluating discriminant validity and convergent validity (Teng et al., 2024), all 24 items that make up the scale were significantly loaded on the target factor, and most of the loadings were greater than 0.3. This indicates that the model has good convergent validity; and all the items’ loadings on non-target factors either did not significantly affect or were lower than their loadings on the target factor, so it can be concluded that the model has good discriminant validity.

**Figure 1:**
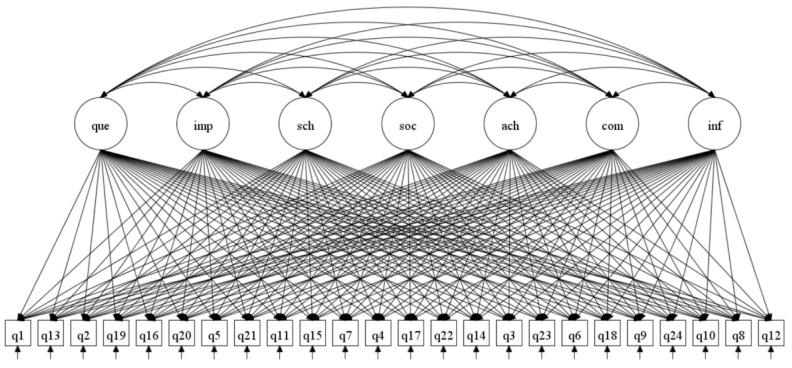
The ESEM measurement model of the framework of EFL teachers’ motivation to pursue an Ed.M. degree.

The ESEM results show that EFL teacher motivation for pursuing an Ed.M. degree includes seven dimensions and the ESEM measurement model and specific item loadings are shown in Figure 1 and Table 4.

In the first factor, there are four items with high loadings in this dimension, which are items 12, 8, 10, and 24 with factor loadings ranging from 0.568 to 0.627. These four items mainly reflect that EFL teachers pursue an Ed.M. degree mainly to obtain academic information, such as cutting-edge news, guidance from experts, and teaching materials, which falls under the category of “academic information acquisition”. It can be seen that EFL teachers have a strong motivation to pursue an Ed.M. degree due to their desire to obtain information resources. Krille (2020) also mentioned that teachers view participating in formal continuous education as an opportunity to refresh and acquire knowledge of different aspects.

The second factor contains five items (items 9, 18, 6, 23, and 3) with high loadings ranging from 0.525 to 0.760. The five items all reflect the need for EFL teachers to pursue an Ed.M. degree for professional competition, such as for career promotion, career development, and recognition from others. They belong to “rival demand”, which is consistent with the connotation of occupational promotion mentioned by Richter et al. (2019), focusing on the positive impact of professional competition on teachers’ continuous learning. It is worth mentioning that item 23 belongs to social responsibility in the original framework, but our verification results show that this item has a high loading of 0.631 under the rival demand dimension. Considering what the item is about (Studying for a master’s degree is an honor for me), we find that EFL teachers pursue an Ed.M. degree to gain honor and also satisfy their inner development needs. This is a psychological level of competition, so it is appropriate to classify this item under the rival demand dimension.

Factor 3 has three items (items 14, 22, and 17) with loadings of 0.553, 0.544, and 0.471, which speaks to teachers’ desire to fulfill academic achievements. It is, thus, called “academic achievement acquisition”, referring to English teachers pursuing an Ed.M. degree to achieve the goal of publishing academic papers, applying for projects, or preparing for future doctoral study. This echoes Tarvid’s (2014) study, which finds one of the aims for teachers to receive graduate education is to write papers and gain new achievements, thus fulfilling personal goals.

In the fourth factor, only two items (Items 4 and 7) have high loadings of 0.914 and 0.757, which belong to “social responsibility”. This mainly refers to EFL teachers’ desire to share their teaching philosophy and cutting-edge information with colleagues in their institution, reflecting their concern about the development of others around them and their desire to promote the progress of colleagues through further studies and repay the institution for their help. This is consistent with the connotation of the community service proposed by Boshier and Collins (1983), which emphasizes that teachers participate in continuous education to serve the development of the community, society, and even humanity. It is worth noting that the scope of social responsibility in this study is not the whole community, the entire society, or human development as previously referred to, but specifically the teaching community of the teachers’ institution.

Four items in the fifth factor have high loadings, which are item 15 (0.801), item 11 (0.752), item 21 (0.723), and item 5 (0.588), respectively, reflecting the motivating effect of institutions’ reputation on EFL teachers’ pursuit of an Ed.M. degree. This factor is named “school context”. Specifically speaking, teachers choose to pursue an Ed.M. degree mainly because the institution where they study is well known in China and there are many nationally renowned academic experts or teaching masters. This is consistent with the accredited university found by Greene et al. (2020), which emphasizes teachers choose to pursue a master’s degree due to factors such as the reputation of the university.

Factor 6 has two items (item 20 and item 16) with high loadings of 0.555 and 0.857, emphasizing the positive impact of support and encouragement from significant others such as friends, colleagues, classmates, and family members on EFL teachers’ motivation to pursue an Ed.M. degree. As found in previous studies, conversations with those who have positive learning experiences will motivate those who want to pursue improvement in their degree (Ajzen, 1991).

Four items in the seventh factor have high loadings, which are item 19 (0.447), item 2 (0.820), item 13 (0.393), and item 1 (0.547), belonging to “cognitive interest”. This factor emphasizes that teachers’ participation in continuous education is driven by intrinsic needs, such as improving their teaching and research abilities, enriching themselves, and fulfilling their long-cherished wishes. Njenga (2023) also mentioned teachers’ participation in continuous education for task performance competence, which means searching for improvements in the roles that teachers take part in.

In addition, McDonald’s omega (ω, also known as CR) is calculated to assess the internal consistency reliability of each factor. In Table 4, most factors’ omegas are above 0.7 except for the factor “academic achievements” (ω = 0.594). However, according to Alamer & Marsh (2022, p. 1485), “compared to CFA, the values of ω tend to decrease in ESEM, and thus researchers should expect and accept this observation as long as other model parameters are comparable”. Thus, this seven-dimensional motivation ESEM model has acceptable internal consistency owing to the good performance in other model parameters (such as factor loadings, and item uniqueness).

In summary, EFL teachers’ motivation to pursue Ed.M. includes academic information acquisition, rival demand, academic achievement acquisition, social responsibility, school context, significant others, and cognitive interest. These findings are consistent with those of Boshier (1991) and Liu (2015) and go a step further by refining and extending new insights into their motivational frameworks among in-service EFL teachers. Firstly, this study contextualizes Boshier’s (1991) framework, which focuses more attention on adult education participants. By employing Boshier’s (1991) framework, this study investigates Chinese EFL teachers’ motivation to pursue Ed.M., thereby enhancing its relevance and applicability. Secondly, both this study and Liu (2015) emphasize the role of internal and external influences in shaping EFL teachers’ decisions to engage in Ed.M. education. But, this study is unique due to its epochal character, especially when China is undergoing a new round of education reform.

**Figure 2:**
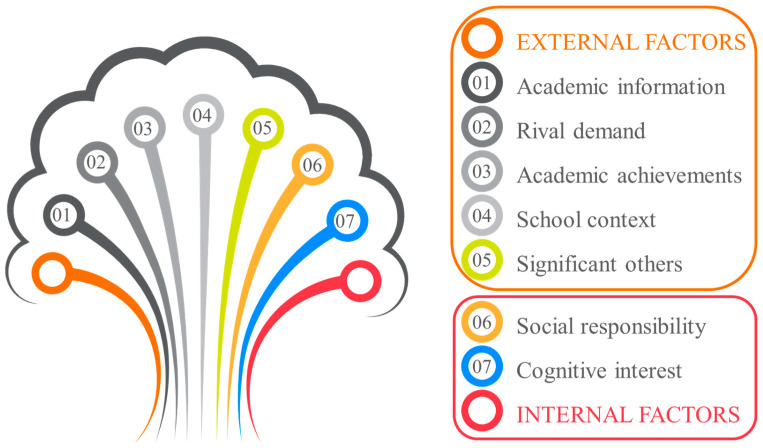
EFL teacher motivation for pursuing Ed.M.

According to Deci and Ryan’s (1985) classification of intrinsic and extrinsic motivation, these seven factors encompass both internal and external factors. Specifically, teachers may seek to enhance their personal abilities and clarify their future development direction through continuous education, demonstrating a sense of social responsibility by contributing to the development of colleagues, the improvement of school research levels, and overall societal development. These factors stem from teachers’ internal needs; thus, cognitive interest and social responsibility are categorized as internal factors. However, teachers may also be inspired by others, or seek advanced teaching theories, research resources, and expert guidance, as well as apply for projects, complete tasks, or seek career advancement, highlighting the important role of external factors in motivating teachers to pursue further education. Therefore, significant others, academic information acquisition, rival demand, academic achievement acquisition, and school context are considered external factors, demonstrating that teachers’ pursuit of an Ed.M. degree is the result of the combined influence of both internal and external factors (see Figure 2).

### 5.2. Levels of EFL Teacher Motivation for Pursuing Ed.M.

As shown in Table 5, the results indicated a high level of EFL teacher motivation (M = 4.36; SD = 0.75). Specifically speaking, EFL teachers are more willing to achieve their goals by means of pursuing Ed.M. Among these motivations, cognitive interest (M = 5.00; SD = 0.72) exerted the highest level, followed by academic information acquisition (M = 4.72; SD = 0.88), rival demand (M = 4.27; SD = 0.86), school context (M = 4.11; SD = 1.07), social responsibility (M = 3.82; SD = 1.14), significant others (M = 3.72; SD = 1.21), and academic achievement acquisition (M = 3.29; SD = 1.05).

This study finds that cognitive interest is the most dominating factor in motivating teachers to pursue an Ed.M. degree, as they are motivated by a desire to improve themselves, both in teaching and research. In addition, they also see continuous education as a long-cherished wish in the process of life development. This is consistent with the findings of Appova and Arbaugh (2018), who believe that the most important driving force for individual development comes from within the individual to meet the desire to improve him/herself. The interview of Teacher C also echoes this finding:

I am pursuing graduate studies largely due to the profound sense that there is still much for me to learn, particularly amidst the continuous deepening of the new round of curriculum reform. Although we receive periodic pieces of training related to this reform, many novel educational concepts remain unclear to me. By pursuing graduate studies, I aspire to gain insights from experts on their unique perspectives of the new curriculum reform, enabling me to keep pace with its advancements and thereby better excel in my teaching endeavors.

(C-I-20230610)

Teacher C feels a sense of uncertainty and confusion regarding the new curriculum reform, grappling with the myriad changes it brought forth. Recognizing the importance of staying abreast with these advancements, he is eager to acquire knowledge and understanding about the reform, aiming to “keep pace with its advancements” and enhance his teaching practices. He believes that by delving into the essential meaning of the new curriculum, he can not only adapt to the changing landscape of education but also elevate the quality of his instruction. With this aspiration in mind, Teacher C embarks on a journey of pursuing an Ed.M. degree, determined to harness the power of knowledge to enrich his teaching endeavors.

In addition, academic information, rival demand, school context, social responsibility, significant others, and academic achievements also affect EFL teacher motivation to pursue an Ed.M. degree to varying degrees. This also illustrates that teachers’ motivation is not the result of a single factor, but rather the combined effects of different factors in their ecological environment (Bronfenbrenner, 1992; Liu, 2024a).

It is noteworthy that academic achievement acquisition scores are the lowest, which can be attributed to multiple complex factors. Firstly, EFL teachers have their daily responsibilities centered around teaching, student management, and school affairs, which consume a significant amount of their time and energy (Alfaifi, 2024). Therefore, when pursuing an Ed.M. degree, they may be more inclined towards enhancing teaching abilities, updating educational ideologies, or obtaining qualifications for career advancement, rather than purely accumulating academic achievements. This career-oriented motivation may weaken their desire to acquire academic achievements. Secondly, acquiring academic achievements often requires extended research and accumulation, and its results may not be immediately seen. In contrast, teachers may prefer that their graduate studies bring immediate professional rewards or significant improvements in teaching effectiveness (Amponsah et al., 2023). This pursuit of immediate benefits may lead teachers to prioritize courses and research projects that can be quickly applied to teaching practice over those that involve lengthy and uncertain academic research. Furthermore, the acquisition of academic achievements is influenced by various external factors such as the academic environment, research resources, and guidance from mentors. For teachers pursuing graduate studies while still working, they may face more complex academic environments and more limited research resources. Additionally, balancing teaching responsibilities and other school affairs may make it difficult for them to obtain sufficient mentorship and support. These factors can further reduce the motivation to acquire academic achievements during graduate studies. Lastly, personal interests, career plans, and academic values can also influence teachers’ motivation to acquire academic achievements through graduate studies. Some teachers may prioritize teaching practice and student growth over pure academic research, or their career plans may not focus primarily on academic achievements. The interview with Teacher D corroborated this point:

Writing articles and applying for research projects do not hold much appeal to me. On one hand, the criteria for professional evaluation and promotion in our province have shifted, with less emphasis placed on publishing papers and securing research grants, and a greater focus on teachers’ achievements in teaching. This change in requirements has naturally influenced my priorities. On the other hand, having already attained a senior professional title, I find myself less inclined to pursue further advancements in that direction. Consequently, the pressure to publish articles or engage in such endeavors has diminished for me. My decision to pursue an Ed.M. degree stems from a genuine desire to update my knowledge and skills and the support from my school. So why not seize this opportunity to broaden my horizons and enrich my educational journey?

(D-I-20230617)

Finally, based on the classification of the above seven motivational factors, this study finds that the level of internal factors (M = 4.61; SD = 0.72) is higher than that of external factors (M = 4.11; SD = 0.70), which is consistent with the opinion that internal factors are the main cause of individual development (Kwakman, 2003; Gorozidis & Papaioannou, 2014).

### 5.3. Differences in EFL Teacher Motivation in Terms of Gender and School Type

To mitigate the effects of the gender imbalance in the sample (male teachers = 60, female teachers = 469), we randomly selected 60 of the female teachers using the SPSS software (version 26.0) and constructed a new sample (male teachers = 60, female teachers = 60). The same was performed with teachers from different types of schools and a new sample (teachers in regular schools = 188, teachers in key schools = 190) was constructed, too.

The results of the independent samples *t*-test revealed significant differences in EFL teacher motivation in terms of gender and school type (see Table 6 and Table 7). Specifically speaking, males perceived higher levels of cognitive interest and rival demand, indicating that males had a stronger desire to improve themselves and to be more competitive when facing more fierce competition. This may be attributed to the fact that males tend to have higher career aspirations than females (Gottfredson, 2002). It is often observed that males, in many societies and cultural contexts, tend to demonstrate a stronger sense of ambition and drive towards their careers compared to females. This phenomenon can manifest in various ways, such as males desiring more demanding jobs to show their competence (Ashby & Schoon, 2010), having higher levels of leadership aspirations (Singer, 1989; Sheppard, 2018), and investing more time and energy into advancing their professional lives (Wolniak et al., 2023). In some cases, males may be more inclined to conduct in-depth research and exploration in a particular field of expertise, which may be related to their stronger cognitive interests. In addition, many male teachers may pay more attention to academic achievements and professional development in their careers. They may prefer to further study their professional fields through graduate studies to broaden their academic horizons and satisfy their thirst for knowledge and academic pursuit.

In the meantime, the competition in the teaching profession is becoming increasingly fierce. Male teachers may be more concerned about their competitive position in their careers, so they are more inclined to pursue graduate studies to enhance their academic qualifications and academic level, thus strengthening their competitiveness in job hunting, job evaluation, and promotion. Female teachers, however, often play multiple roles in the family (Sheppard, 2018), including being a wife, a mother, and so on. These factors may be obstacles to their motivation to pursue graduate studies.

In addition, compared to those in key schools, the teachers in regular schools exhibited a stronger motivation to pursue an Ed.M. degree due to the influence of significant others in their lives (see Table 7). This disparity can stem from several factors:

The first one is the resource and support environment. Key schools often possess richer educational resources and more comprehensive support systems, including higher salaries, advanced teaching facilities, and ample opportunities for professional development (J. Li, 2022). In such environments, teachers may rely more on internal resources and opportunities within the school for self-improvement, feeling less compelled by external influences from significant others to pursue graduate studies. In contrast, teachers in regular schools, faced with relatively limited resources, may depend more on external support and encouragement, including from family members, friends, or senior colleagues, to motivate them to further their education.

The second one is the differences between career competition and pressure. Teachers in key schools may enjoy greater job satisfaction and social status (Liu & Chu, 2022; Liu et al., 2024b). This sense of stability and fulfillment in their careers may to some extent diminish their urgency to pursue graduate studies. However, teachers in regular schools may undergo greater job pressures and uncertainties, making them more inclined to use graduate studies to enhance their competitiveness and increase opportunities for career development. Therefore, they may be more susceptible to the influence of significant others advocating the benefits of pursuing graduate studies.

### 5.4. Implications

To enhance the motivation of EFL teachers to pursue an Ed.M. degree and actively engage in continuous education and self-improvement, some suggestions are proposed to stimulate and sustain teachers’ motivation to pursue continuous studies.

Firstly, teachers ought to foster a wholesome perspective and a mindset toward graduate studies, emphasizing the paramount importance of embarking on an Ed.M. degree. It is understandable that teachers, weary from the rigors of daily instruction, yearn for a break during holidays. However, pursuing an Ed.M. degree necessitates dedicating leisure hours, potentially diminishing opportunities for rejuvenation. This reality may tempt some to perceive the endeavor as a means of escaping routine or alleviating working stress. Yet, it is imperative for these teachers to introspect deeply on the significance of pursuing an Ed.M. degree, which is crucial to prevent time from being squandered during periods of intense study. In addition, teachers should anticipate challenges that lie ahead, make psychological preparations before enrollment, and be resilient agents (Liu, 2024b). For example, they can gain an understanding of graduation requirements in advance to weigh whether they will make it. By adjusting their mindset and ensuring optimal mental and emotional well-being (Liu et al., 2024c), they can approach their studies with renewed vigor, recognizing that further education is a transformative journey, not merely a reprieve from daily duties.

Secondly, educational institutions where teachers work must provide sufficient support. When possible, they should also reduce teachers’ workloads (Liu et al., 2024a) to give them the time and energy needed to focus on their studies. Furthermore, institutions can establish special scholarships to encourage in-service teachers to pursue an Ed.M. degree and implement policies that recognize academic qualification enhancement as a key factor in professional title promotion. When teachers feel valued and supported by their institutions, they are more likely to remain after graduation, contributing to the school’s long-term growth and development. Institutional support not only helps teachers manage their academic responsibilities alongside their teaching duties but also fosters a sense of belonging, strengthening their commitment to their careers. At the same time, teachers must take an active role in their development by refining their skills, setting clear goals, and managing their time effectively. This balance is essential for achieving both professional and academic excellence while ensuring high-quality instruction.

Lastly, the government ought to enact policies that actively support teachers in their pursuit of graduate studies, with a particular emphasis on financial assistance (Ehrenberg, 1992; Wang & Fu, 2019). By alleviating financial constraints, these measures enable educators to embark on their academic journeys with tranquility, unburdened by monetary worries. This approach has already been successfully adopted in various regions. For instance, in Dongguan City, China, schools or the government provide subsidies covering tuition fees, transportation, and other related expenses (Dongguan Education Bureau, 2019). Such benefits significantly reduce teachers’ financial burdens, freeing up resources for professional development, academic exchanges, and personal growth. Furthermore, some cities have introduced incentives for teachers upon completion of their studies, including talent subsidies, preferential housing rates, and travel allowances. These policies not only serve as tangible rewards but also act as potent motivators, encouraging teachers to commit fully to their studies and strive for excellence. Ultimately, such comprehensive support systems will cultivate a culture of continuous learning and professional advancement within the teaching profession.

## 6. Conclusions

This study applied Boshier’s (1991) and Liu’s (2015) motivation frameworks to examine EFL teachers’ motivation for pursuing an Ed.M. degree. The findings confirm that this motivation is multidimensional, involving factors such as cognitive interest, academic information, rival demand, school context, social responsibility, significant others, and academic achievements. Additionally, this study adapted these motivation frameworks to the context of Chinese EFL teachers. It contributes to previous research by deepening the understanding of teacher motivation in this area. The results also highlight differences in motivation based on gender and school type, underscoring the complexity of teacher motivation. These insights provide a comprehensive view of EFL teachers’ motivation to pursue an Ed.M. degree. They can help stakeholders develop strategies to support teachers’ personal and professional growth in education.

This study has several limitations that should be acknowledged. First, the sample size was relatively modest and primarily drawn from a limited number of regions in China. As a result, the findings may not be fully generalizable to EFL teachers across different regions or international teaching contexts. To improve representativeness, future research should include a more diverse sample, incorporating teachers from various geographical locations in China as well as those working in international settings. This would provide a more comprehensive understanding of the factors influencing their motivation to pursue an Ed.M. degree. Second, while this study primarily employed a quantitative research design, qualitative methods were only used in a limited capacity and did not constitute a fully developed case study. This constraint makes it difficult to capture the complex, interactive mechanisms underlying teachers’ motivations for graduate study. Future research should adopt more in-depth qualitative approaches, such as longitudinal case studies or in-depth interviews, to gain richer insights into the underlying motivational dynamics. Lastly, this study did not examine the long-term impact of obtaining an Ed.M. degree on teachers’ professional development and instructional practices. Future research could explore the experiences, challenges, and career trajectories of EFL teachers who have completed their Ed.M. programs. Investigating how this advanced degree influences their teaching practices, job satisfaction, and career advancement would provide valuable insights for policymakers and educators seeking to support teacher professional development more effectively.

## Figures and Tables

**Table 1:** Different versions of Boshier’s EPS.

Boshier (1971)	Boshier (1977)	Boshier and Collins (1983)
other-directed professional advancement	professional advancement	professional advancement
educational preparedness	cognitive interest	cognitive interest
self-centredness versus altruism	social welfare	community service
social contact	——	social contact
——	external expectations	external expectations
——	escape/stimulation	social stimulation

**Table 2:** The information of participants in the questionnaire.

Demographic Variable	Category	Value	Overall Population Statistics ^a^
Gender; *N* (%)			*χ*^2^ = 3.879 (*p* = 0.05)
	Male	61 (11.5%)	220,379 (14.5%)
	Female	468 (88.5%)	1,294,137 (85.5%)
School location; *N* (%)			Not available
	Urban area	333 (62.9%)	
	Town area	178 (33.6%)	
	Rural Area	18 (3.4%)	
School Type; *N* (%)			Not available
	Key school	342 (64.7%)	
	Regular school	187 (35.3%)	

^a^: Data from the Ministry of Education of the People’s Republic of China http://www.moe.gov.cn/jyb_sjzl/moe_560/2022/ (accessed on 9 March 2025).

**Table 3:** Descriptive analysis of the survey.

Items	Mean	SD	Kurtosis	Skew
an1	4.62	1.17	0.28	−0.74
an2	5.18	0.82	−0.16	−0.69
an3	4.39	1.14	−0.02	−0.44
an4	3.94	1.21	−0.18	−0.24
an5	4.14	1.25	−0.13	−0.44
an6	4.55	1.18	0.38	−0.77
an7	3.70	1.21	−0.28	−0.17
an8	4.70	1.14	0.53	−0.83
an9	4.82	1.02	0.97	−0.85
an10	4.89	0.94	0.86	−0.78
an11	4.27	1.18	0.04	−0.5
an12	4.53	1.24	0.47	−0.83
an13	5.13	1.04	3.28	−1.58
an14	3.71	1.31	−0.38	−0.23
an15	4.16	1.27	−0.12	−0.51
an16	3.96	1.43	−0.58	−0.42
an17	2.92	1.55	−0.84	0.36
an18	3.36	1.38	−0.64	−0.07
an19	5.05	0.91	0.58	−0.83
an20	3.49	1.42	−0.71	−0.11
an21	3.88	1.36	−0.55	−0.27
an22	3.25	1.30	−0.33	0.10
an23	4.22	1.29	−0.24	−0.49
an24	4.76	1.05	0.69	−0.81

**Table 4:** Results of ESEM for the seven-dimensional motivation framework.

Excerpts of Items	Factor 1	Factor 2	Factor 3	Factor 4	Factor 5	Factor 6	Factor 7	Item Uniqueness
	Academic Information	Rival Demand	Academic Achievements	Social Responsibility	School Context	Significant Others	Cognitive Interest	
Q12 To get acquainted with renowned academic experts	0.602 *	0.034	0.015	0.059	0.098 *	−0.021	0.163 *	0.398
Q8 To be guided by renowned academic professionals	0.617 *	−0.039	0.092	0.111 *	0.122 *	−0.018	0.119 *	0.374
Q10 To gain more academic and teaching resources	0.568 *	0.095 *	0.224 *	0.083 *	−0.028	−0.067	0.208 *	0.350
Q24 To be guided by renowned teaching masters	0.571 *	−0.006	−0.046	0.023	0.232 *	0.025	0.170 *	0.360
Q9 To strengthen my qualifications for future professional certifications	0.225 *	0.653 *	0.048	−0.031	−0.165 *	0.082	−0.104 *	0.525
Q18 To be highly thought of by others	−0.241 *	0.766 *	0.185 *	−0.155 *	0.103 *	−0.044	−0.077	0.352
Q6 To increase my chips for future career development	0.153 *	0.692 *	−0.093	0.020	−0.063	0.047	−0.03	0.497
Q23 To be honored and respected	−0.037	0.631 *	−0.017	−0.021	0.131 *	0.038	0.161 *	0.420
Q3 To gain more prestige among my students	−0.162 *	0.515 *	0.039	0.245 *	0.019	−0.067	0.248 *	0.502
Q14 To publish high-quality papers	0.097 *	0.091 *	0.553 *	−0.046	0.050	0.055	0.032	0.576
Q22 To apply for a project	0.121 *	0.193 *	0.544 *	0.142 *	0.129 *	0.070	−0.183 *	0.387
Q17 To prepare for the doctoral qualifying examination	−0.004	−0.046	0.471 *	−0.088 *	0.090	0.069	0.133 *	0.718
Q4 To share updated teaching ideas with my colleagues	0.018	0.001	0.014	0.914 *	0.005	−0.003	0.098 *	0.070
Q7 To share academic information with my colleagues	0.110 *	0.008	0.002	0.757 *	0.137 *	0.121 *	−0.083 *	0.240
Q15 To study from prestigious teachers	0.094 *	−0.118 *	0.156 *	−0.019	0.801 *	0.063 *	0.043	0.190
Q11 To get acquainted with renowned academic experts	0.154 *	−0.061 *	0.164 *	0.067 *	0.752 *	−0.030	0.009	0.214
Q21 To study in a prestigious major	0.023	0.145 *	−0.028	−0.016	0.723 *	0.076 *	−0.049	0.358
Q5 To study at a prestigious university	−0.055	0.187 *	−0.101 *	0.184 *	0.588 *	0.044	0.085 *	0.362
Q20 To comply with the encouragement of my friends, colleagues, and classmates	−0.021	0.057	0.101 *	0.222 *	−0.068	0.555 *	−0.071	0.566
Q16 To comply with the encouragement of my family	−0.131 *	−0.043	0.011	−0.094 *	0.055	0.857 *	0.117 *	0.264
Q19 To improve my teaching ability	0.294 *	0.193 *	−0.061	−0.046	0.064	0.056	0.447 *	0.419
Q2 To improve my ability to teach and research	0.105 *	−0.039	0.132 *	0.071 *	−0.192 *	0.055	0.82 *	0.281
Q13 To secure self-improvement	0.040 *	0.080	−0.181 *	−0.083 *	0.089 *	0.106 *	0.393 *	0.421
Q1 To fulfill my long-cherished wish	−0.203 *	0.063	−0.042	0.08 *	0.274 *	0.007	0.547 *	0.521
Reliability (CR)	0.790	0.822	0.594	0.795	0.879	0.706	0.748	

* *p* < 0.05. Bolden figure: the item primarily loaded on the factor.

**Table 5:** The levels of EFL teacher motivation for pursuing Ed.M.

	Min	Max	M	SD
cognitive interest	1.75	6.00	5.00	0.72
academic information	1.25	6.00	4.72	0.88
rival demand	1.00	6.00	4.27	0.86
school context	1.00	6.00	4.11	1.07
social responsibility	1.00	6.00	3.82	1.14
significant others	1.00	6.00	3.72	1.21
academic achievements	1.00	6.00	3.29	1.05
external factors	1.44	5.83	4.11	0.70
internal factors	1.83	6.00	4.60	0.72
global motivation	1.63	6.00	4.36	0.75

**Table 6:** Differences in EFL teacher motivation in terms of gender.

	Male (*N* = 60)		Female (*N* = 60)		MD	*t*	*df*	*p*
	M	SD	M	SD				
cognitive interest	4.97	0.79	4.11	0.64	0.86	6.522	118	0.013
rival demand	4.14	0.98	3.85	0.78	0.29	4.714	118	0.048

**Table 7:** Differences in EFL teacher motivation in terms of school type.

	Regular (*N* = 188)		Key (*N* = 190)		MD	*t*	*df*	*p*
	M	SD	M	SD				
significant others	3.75	1.27	3.60	1.10	0.125	−1.016	375	0.028

## Data Availability

The raw data supporting the conclusions of this article will be made available by the authors upon request.

## Abbreviations

EFL	English as a Foreign Language
Ed.M.	Master of Education
EPS	Education Participation Scale
ESEM	Exploratory Structural Equation Modeling
MAED	Master of Arts in Education
